# Hearing and sociality: the implications of hearing loss on social life

**DOI:** 10.3389/fnins.2023.1245434

**Published:** 2023-10-03

**Authors:** Archana Podury, Nicole T. Jiam, Minsu Kim, Jonah I. Donnenfield, Amar Dhand

**Affiliations:** ^1^Harvard Medical School, Boston, MA, United States; ^2^Department of Otolaryngology-Head and Neck Surgery, Massachusetts Eye and Ear, Boston, MA, United States; ^3^Department of Neurology, Brigham and Women’s Hospital, Boston, MA, United States; ^4^Department of Otolaryngology-Head & Neck Surgery, University of California, San Diego, San Diego, CA, United States; ^5^Department of Molecular and Cellular Biology, Harvard University, Cambridge, MA, United States

**Keywords:** hearing loss, cochlear implantation, social networks, social isolation, health outcomes, neural plasticity

## Abstract

Hearing is essential to the formation of social relationships and is the principal afferent of social life. Yet hearing loss, which is one of the most prevalent forms of sensory disability worldwide and is critical for social development, has received little attention from the social interventionalist perspective. The purpose of this mini-review is to describe the basic neurobiological principles of hearing and to explore the reciprocal relationships between social support, hearing loss, and its psychosocial comorbidities. We also discuss the role of social enrichment in sensorineural recovery and identify open questions within the fields of hearing physiology and social networks.

## Introduction

Throughout human history, communication has played a central role in shaping our social world. Language enabled our ancestors to share information and coordinate activities to facilitate social cohesion. As societies advanced, language became more complex, allowing for the transmission of ideas across generations and the construction of collective identities (Carvalho et al., 2016). Across time, hearing has remained integral to the passage of rich interpersonal information and may be thought of as the principal afferent system of social life.

Hearing plays multiple essential roles in shaping our social interactions. First, hearing is essential for afferent and efferent functions of language. Auditory input in early childhood drives the maturation of white matter microstructure in brain areas related to speech comprehension and production. Moreover, hearing continues to be important throughout life for features of communication such as speech enunciation (Cheng et al., 2019). Second, the acoustic features of communication carry rich social information that can allow a listener to orient to the speaker, extract meaning within lexical languages, and attribute emotional valence through cues, such as tone and tempo (Coutinho and Dibben, 2013). Audition is often more important than other sensory modalities, such as vision, to interpret emotional cues in communication (Valente et al., 2012; Picou et al., 2018). Finally, hearing affords volume modulation that allows for directed communication in a hushed voice to an intimate or loud voice in a crowd. A speaker can modulate the size of the social group receiving information through such use of auditory cues.

Hearing loss, therefore, has multifold effects on social connectedness, yet has received little attention from the social interventionalist perspective. Hearing loss is a highly prevalent sensory disability affecting nearly 470 million people worldwide, and this number is expected to grow to 900 million by 2050 as our population continues to age (Davis and Hoffman, 2019). Individuals with hearing loss are at increased risk for medical comorbidities, such as dementia, falls, and cognitive impairment (Cunningham and Tucci, 2017; GBD 2019 Hearing Loss Collaborators, 2021). Hearing loss also may have significant psychosocial impacts, ranging from social isolation and depression to increased risk of psychosocial disability and falls, which may in part mediate its medical comorbidities (Shukla et al., 2020, 2021). Over the course of an individual’s lifetime, hearing loss may have social profound implications, from difficulties with social development in early childhood to compounded morbidity and isolation in older adults.

**Figure 1:**
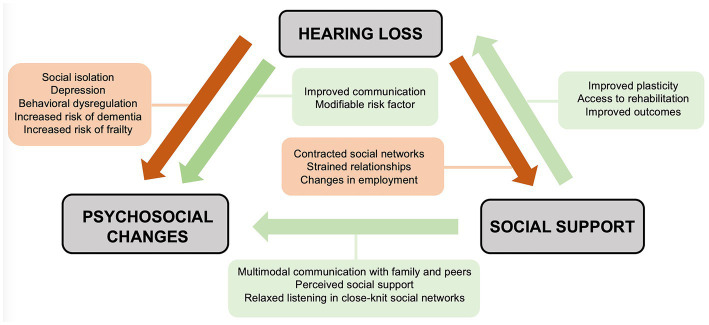
Social interventionist approach detailing the reciprocal relationships between hearing loss, psychosocial comorbidities, and social support.

Just as hearing loss may shape one’s social world, the social environment may in turn influence individuals’ experiences with hearing loss (Figure 1). Individuals with hearing loss demonstrate wide variation in psychosocial outcomes (Polat, 2003). Many individuals will adopt varied approaches to support communication needs, such as multimodal communication styles, hearing assistance devices such as cochlear implants and hearing aids, and social support networks within deaf and hard of hearing (DHH) communities (Pray and Jordan, 2010). Additionally, one’s response to rehabilitation after the introduction of a hearing assistance device is closely related to social support (Gao et al., 2020). These observations highlight the importance of a social interventionalist framework that studies the reciprocal relationship between hearing loss and one’s social world.

### Neurobiology of hearing

Sound is a pressure transduction wave that is converted from a mechanical stimulus into an electrical stimulus by the ear. Sound waves are transduced into vibrations, starting with the tympanic membrane of the external ear and the ossicles of the middle ear. These vibrations are transmitted to the inner ear via the oval window, which generates a fluid wave within the endolymph-filled cochlea. This fluid wave bends tip links of stereocilia and opens mechanically gated ion channels on hair cells, the sensorineural tissue lining the cochlea. Surrounding potassium-rich endolymph depolarizes the hair cells and generates action potentials, which are transmitted via the auditory nerve to the brainstem and auditory cortex. Hair cells are organized tonotopically along the length of the cochlea, and the location of activation corresponds to the perceived frequency of sound (Fettiplace, 2017).

Disruptions to the conductive apparatus, including the tympanic membrane, ossicles, or bony labyrinth, result in conductive hearing loss. Disruptions to the sensorineural tissue, including hair cells within the cochlea or the auditory nerve, result in sensorineural hearing loss. Hearing loss involving both mechanisms is characterized as mixed hearing loss (Anastasiadou and Khalili, 2023).

Etiologies of hearing loss are multifold. Up to 50% of cases have a known genetic etiology. Genetic causes of hearing loss may present in childhood (USH1, USH2, KCNQ1, KCNE1), with progressive onset into late adulthood (Connexin 26, MYO15A, STRC, TMC1, KCNQ4), or during varied points in an individual’s lifetime (SLC26A4, COL4A5, USH3) (Shearer et al., 1999; Boeckhaus et al., 2020; Young and Ng, 2023). Genetic syndromes vary in degree of cochlear dysplasia and extracochlear involvement. Prenatal exposures, such as bacterial and viral infections or teratogens, account for up to 20% cases and can lead to onset of hearing loss during infancy or early childhood. Various other exposures may precipitate hearing loss later in life, including ototoxic agents, viral meningitis, trauma, and recurrent otitis media. Progressive degeneration of the hair cells over one’s lifetime, known as presbycusis, can result in sensorineural hearing loss in up to two-thirds of the aged (Harley et al., 2010).

Rehabilitation options vary by the mechanism of hearing loss. Patients may benefit from amplification of sound through hearing aids, or from assisted listening devices such as loops, FM, infrared, or signaling systems (Michels et al., 2019). Cochlear implantation is a widely performed and efficacious intervention for hearing loss and requires an intact cochlear nerve. Where hearing aids have limited benefit, conductive hearing loss or profound unilateral sensorineural hearing loss may be addressed with bone conduction devices or middle ear implants. For etiologies with compromised integrity of the cochlear nerve, patients may benefit from implants that directly stimulate the brainstem. Many interventions involve a period of rehabilitation where patient outcomes are measured by the ability to detect sound and discriminate speech. Communication with others is an important endpoint of successful rehabilitation (Szathmáry and Számadó, 2008).

### Neurobiology of language

The neurobiology of hearing is intrinsically coupled to communication. Language involves shared attention to detect, discriminate, and extract meaning from vocalizations, which requires auditory processing (Mueller et al., 2012). Electrophysiological measures of auditory perception suggest that the ability to extract linguistic rules develops during early infancy: newborns are sensitive to transitional probabilities in syllables from their mothers’ spoken language, and 3-month-old infants can identify rule violations within patterns of auditory cues (Chomsky and Skinner, 1959; Flo et al., 2022). Infants preferentially attend to auditory information over visual information compared to adults, suggesting the importance of auditory input in shaping early language skills (Chomsky, 1965). When infants are deprived of auditory stimuli, they exhibit delayed language development and communication skills (Purves et al., 2001).

One of the earliest explanations of language acquisition is B. F. Skinner’s Behaviorist theory. Behaviorists view language as a product of imitation and reinforcement: successful associations of words are positively reinforced with reward (Vygotsky, 1980). Noam Chomsky proposed the Nativist theory, which argues that children are born with innate abilities to acquire language based on “universal grammar” (Hurford, 1991; Yang et al., 2017). Nativist theorists believe that in order to activate this innate ability, children need environmental exposure before the critical period for language acquisition (from birth to 2 years) (Koelsch et al., 2018). The social pragmatist view purports that language develops through a socially-mediated process in which a child’s linguistic skill is heavily dependent on social engagement. These different theories of language development suggest that the neurobiology of speech and an individual’s social environment are interrelated.

Neuroanatomy studies suggest that the auditory cortex may play a central role in multimodal social processing. Functional magnetic resonance imaging (fMRI) identifies connections from the primary auditory cortex to limbic/paralimbic, visual, somatosensory, and motor regions - many of which participate in emotional processing and reinforcement of motor sequences (Koelsch et al., 2018). These multimodal networks are activated when participants conjure social scenarios in response to auditory stimuli, such as imagining “people partying” during joyful music. The auditory cortex also receives corticofugal projections from the prefrontal cortex, which are thought to contain Bayesian priors that modulate perception of sound based on previous social experience (Asilador and Llano, 2021). These projections are dynamic and evolve in parallel with linguistic needs (Robinson and Sloutsky, 2004; Song et al., 2017).

### Hearing loss and social development in childhood

Given the importance of auditory processing, hearing loss is important to address in early childhood as its presentation may influence linguistic and social development. Several studies, summarized in Table 1, describe psychosocial effects hearing loss may have throughout early childhood, adolescence, adulthood, and older age.

**Table 1:** Hearing loss and psychosocial development.

Early childhood	
Delayed language abilities	Vygotsky (1980), Schneider et al. (2010), Houston et al. (2012), Sandmann et al. (2012), Friedmann and Rusou (2015), Netten et al. (2015), Anderson et al. (2017), Kwok et al. (2018)
Varied emotional regulation	Hurford (1991), Schorr et al. (2009), Wiefferink et al. (2012), Rolfe and Gardner (2016), Yang et al. (2017), Michels et al. (2019)
Disrupted social engagement and psychosocial development	Hurford (1991), Khan et al. (2005), Houston et al. (2012), Wiefferink et al. (2012), Friedmann and Rusou (2015), Rolfe and Gardner (2016), Wong et al. (2017), Lieu et al. (2020)
*Adolescence*	
Lower reported quality of life	Robinson and Sloutsky (2004), Schorr et al. (2009), Loy et al. (2010), Kushalnagar et al. (2011)
Diminished social well-being	Percy-Smith et al. (2008), Schorr et al. (2009), Kushalnagar et al. (2011), Kwok et al. (2018)
Social isolation: difficulties in peer and familial relationships	Huber et al. (2015), Koelsch et al. (2018), Lieu et al. (2020), Zaidman-Zait and Most (2020)
Increased psychosocial risk: behavioral dysregulation	Polat (2003), Netten et al. (2015), Monshizadeh et al. (2018), Sarant et al. (2018), Asilador and Llano (2021)
*Adulthood*	
Diminished social well-being	Monzani et al. (2008), Cunningham and Tucci (2017), Monshizadeh et al. (2018), Boerrigter et al. (2019), Niazi et al. (2020)
Social isolation: interpersonal	Graaf and Bijl (2002), Cunningham and Tucci (2017), Ellis et al. (2021), Shukla et al. (2020, 2021)
Social isolation: structural	Wiefferink et al. (2012), Shan et al. (2020), Shukla et al. (2021)
Increased psychosocial risk: depression and anxiety	Huber et al. (2015), Cunningham and Tucci (2017), Monshizadeh et al. (2018), Niazi et al. (2020), Dobrota et al. (2022)
*Older age*	
Cognitive decline and dementia	Cunningham and Tucci (2017), Cheng et al. (2019), Idstad and Engdahl (2019), GBD 2019 Hearing Loss Collaborators (2021), Yeo et al. (2022), Anastasiadou and Khalili (2023)
Falls and frailty	Jiam et al. (2016), Moser et al. (2017), Cheng et al. (2019), GBD 2019 Hearing Loss Collaborators (2021)
Increased psychosocial risk: depression and anxiety	Holt-Lunstad et al. (2010), Moser et al. (2017), Tang et al. (2017), Cheng et al. (2019), Michels et al. (2019), Gao et al. (2020), GBD 2019 Hearing Loss Collaborators (2021)

Children aged 1 to 5 years who have not yet received cochlear implants or are newly implanted have been shown to adopt different emotional regulation behaviors compared to hearing children. This difference may be in part due to absent pitch information required for detecting voice emotion (Netten et al., 2015). Interestingly, Schorr et al. (2009) found that scores on a voice emotion recognition test better predicted quality of life in children with hearing loss than word recognition scores (Schorr et al., 2009). Cochlear implants provide some pitch information that may help DHH children detect voice emotion and engage more fully in social interactions, highlighting the importance of early support for hearing loss (Wiefferink et al., 2012).

Children with hearing loss may also face increased psychosocial difficulties, such as behavioral dysregulation and social isolation, in settings with hearing children (Robinson and Sloutsky, 2004; Khan et al., 2005; Anmyr et al., 2015; de Moura et al., 2020). A cluster analysis of 140 adolescents with cochlear implants showed a significantly higher number of hyperactivity and conduct problems in cochlear implant users who had challenges understanding speech in noise compared to cochlear implant users with good speech perception performance or normal hearing adolescents (Huber et al., 2015). This difference may be mitigated in those who have received timely cochlear implants with auditory verbal rehabilitation (Monshizadeh et al., 2018). These social challenges can have profound implications; a cohort study of 48,606 participants found that children with hearing loss were half as likely to pursue higher education (Idstad and Engdahl, 2019).

Some studies suggest that psychosocial difficulties may be related to adolescents’ ability to communicate with parents, peers, and other members of their social environment, beyond just sensory discrimination or linguistic ability (Loy et al., 2010). Polat (2003) found that in a cohort of 1,097 DHH students, psychosocial adjustment was positively related to HA use, speech intelligibility, and school communication methods (Polat, 2003). Receptive language ability, high parent involvement, peer support, and communication mode at home are associated with of fewer psychosocial problems in adolescents with cochlear implants (Kushalnagar et al., 2011; Sarant et al., 2018). Collectively, these findings suggest that social environment, rather than sensory disability itself, may be an important mediator of the psychosocial impacts of hearing loss in children.

### Hearing loss and social life in adulthood

Adult-onset hearing loss is associated with various social and cognitive challenges that may increase morbidity in older adults. Hearing loss later in life may result in isolation from social settings, including group dining and places of employment, and contribute to strain within interpersonal relationships (Shan et al., 2020). Impaired auditory processing can make conversations more difficult to follow in noisy environments and increase cognitive load, which may lead to frustration and avoidance of social activities (Monzani et al., 2008). As a result, DHH adults often have weaker social networks and more depressive symptoms compared to hearing adults (Niazi et al., 2020; Dobrota et al., 2022). Degree of hearing loss has been associated with unemployment, workplace fatigue, and increased need for sick leave (Emmett and Francis, 2015; Svinndal et al., 2018). Hearing loss also has implications on DHH individuals’ relationships with their community. Patients with mild to severe hearing loss show up to 80% increased reliance on formal or informal social support, and DHH individuals with unaddressed hearing loss are twice as likely to rely on community support services (Yeo et al., 2022).

In elderly patients, hearing loss can have significant consequences with compounded morbidity. A recent meta-analysis found that hearing loss was associated with cognitive decline, including Alzheimer’s disease and dementia (Jiam et al., 2016). Though the mechanisms are not understood, the study also found that hearing loss is associated with increased risk of physical morbidity, including falls, frailty, and impaired recovery after neurological injury (Fang et al., 2019; Gao et al., 2020). One possibility is that older adults with hearing loss may have reduced communication with social networks, and activation of social networks during injury is an important prognostic factor for timely intervention (Dhand et al., 2016; Zachrison et al., 2019). Another possibility is that cognitive changes related to hearing loss may directly increase the risk for future physical injury. Finally, social isolation itself is an independent risk factor for morbidity, and stronger social networks are associated with 50% increased likelihood of survival (Holt-Lunstad et al., 2010). As nearly two-thirds of older adults develop hearing loss, these mechanisms have important implications for the social and physical wellbeing of the aged and constitute an ongoing area of study.

While hearing loss in adulthood can have wide-ranging psychosocial consequences, adults with robust support networks show significantly fewer adverse outcomes and some pro-social benefits (Moser et al., 2017; Dunn et al., 2021). Dunn et al. (2021) found that social isolation during the COVID-19 pandemic allowed for a relaxed listening environment with fewer speakers, with CI users reporting lower levels of anxiety resulting from hearing difficulties compared to pre-COVID (Dhand et al., 2022). These findings suggest that hearing loss resulting in exclusion from social networks may lead to detrimental psychosocial and cognitive effects, whereas closely-knit networks that support communication may be protective and even beneficial for DHH adults.

### Social support and sensorineural recovery

An individual’s social environment may influence his or her response to hearing loss interventions. One such example is recovery of sensory function after cochlear implantation.

Neural plasticity is essential for recovery from sensory disability. This principle is highlighted in models of rehabilitation after acute neurological injury, such as stroke. Following the onset of stroke, activation of plasticity-responsive sensory inputs is necessary to restore multimodal function. Environmental enrichment has been shown to improve recovery of sensory function after acute neurological injury (Praag et al., 2000; Rivera et al., 2020). Environmental enrichment typically consists of social engagement, novel stimuli, and exercises that target functional deficits following injury. Models of stroke rehabilitation coupled with environmental enrichment have been associated with synaptogenesis, increased dendritic and axonal remodeling, upregulation of growth-promoting factors (BDNF, Gap43, FGF-2), and increased sensitivity to new sensory input within the perilesional cortex (Carmichael et al., 2005; Vermeire et al., 2008; Murphy and Corbett, 2009; Schwengel et al., 2016). It is important to note that while social engagement is an essential component of environmental enrichment, other factors, such as novel stimuli and exercise, may contribute to improved plasticity and post-stroke recovery.

Effective response to cochlear implantation similarly requires neural plasticity within primary auditory and associative cortices. This idea is highlighted by differences in functional imaging studies in congenitally deaf and post-lingually deafened cochlear implant users. For instance, post-lingually deafened patients show tonotopic reorganization of the auditory cortex toward newly perceived frequencies following cochlear implant activation (Graaf and Bijl, 2002). This reorganization is minimally observed in pre-lingually deafened patients, who have not had as much experience with speech in association with hearing. It is possible that pre-lingually deafened patients have reduced capacity for plasticity within the auditory cortex (McKay, 2018). Though empiric studies are limited, principles of environmental enrichment may extend to the recovery of auditory function following cochlear implantation, where social support and multimodal communication may provide an enriched environment for neural plasticity (Robinson, 1998; Lomber et al., 2010). This area constitutes a gap in the literature that merits further investigation.

## Discussion

Given the varied etiologies of hearing loss, it is important that social interventions are tailored to the unique needs of each group. For young children with hearing loss, early intervention is critical to attain near-normal language abilities. Various social determinants of health, such as race, insurance status, or residency in a rural area, may delay diagnosis of hearing loss and receipt of cochlear implantation services. Interventions must address geographic and socioeconomic barriers (Lee et al., 2001; Han et al., 2019). For pediatric patients where interventions involve shared decision-making with the child’s support system, surgeons must be cognizant of factors that influence family decision-making, including language barriers, comfort with healthcare utilization, cultural practices, and community perspectives on hearing loss (Schneider et al., 2010; Sandmann et al., 2012; Anderson et al., 2017). As social support significantly influences adherence to listening devices, it is important to council caregivers on long-term behavioral reinforcement and provide resources to mitigate caregiver burnout.

DHH children may face challenges in environments with hearing children, and adverse outcomes can be mitigated with robust peer and familial support networks. Parental support and closeness of social networks in school are associated with improved quality of life in children with hearing loss (Houston et al., 2012; Friedmann and Rusou, 2015; Rolfe and Gardner, 2016). Of children with similar speech discrimination scores, those who reported better ability to communicate at school and home also reported higher quality of life. Rehabilitation programs that incorporate peer group engagement are especially effective at improving communication ability and quality of life in cochlear implant users (Kwok et al., 2018; Lieu et al., 2020). These findings are especially important for children who are not candidates for hearing assistance devices, as effective communication within support networks without attainment of near-normal speech of language scores may still protect against adverse outcomes.

Adult-onset hearing loss can be challenging as patients may struggle to adapt to new communication strategies and may regard changing social dynamics with frustration or shame (Wong et al., 2017). A survey found that the majority of patients at an audiology clinic, particularly older or retired individuals, were persuaded to seek care by a family member; a minority of patients were self-motivated (Khan et al., 2005). This phenomenon coincides with a wealth of network science literature that suggests that activation of social networks during stroke or cardiovascular injury is a primary determinant in seeking care. Quantitative features of patients’ social networks, such as size and structure, predict time to arrival at hospital and long-term health outcomes (Percy-Smith et al., 2008; Zaidman-Zait and Most, 2020). Social networks constitute a growing field of study with multifold applications to patients with hearing loss.

Cochlear implant users with similar performance metrics may experience different degrees of cognitive load, which can drive social avoidance. Pupillometry is a growing area of study that assesses listening effort in adult cochlear implant recipients, where pupil diameter may distinguish tasks that are more or less effortful (Cardemil et al., 2014; Anmyr et al., 2015; Gao et al., 2020). Other phenomena with poorly-understood mechanisms, such as auditory overstimulation, may also contribute to exhaustion cochlear implant use and social isolation. Further studies on the functional neuroanatomy of hearing may provide avenues for rehabilitation including multimodal rehabilitation, technology-assisted interactions, and neural modulation (Mahoney et al., 1996; de Moura et al., 2020).

To better understand the social dynamics of patients with hearing loss, it is important explore new methods of acquiring social data beyond self-report (West, 2017). Wearable devices can quantify real-time interactions with social partners. One application is SocialBit, an algorithm which detects users’ daily auditory interactions and characterizes them as social or non-social based on acoustic features (Ellis et al., 2021). The algorithm is currently being trained on patients with diverse communication needs, including those with aphasia, stroke, and dementia, and may be applied to individuals with hearing loss. Similar audio recorders have been used to monitor interactions between conversational partners but have yet to be applied to clinical contexts (Dhand et al., 2022). Real-time social data is critical to monitor changes to one’s social network and design social interventions.

## Conclusion

Hearing plays an important role throughout life in language development, communication, and forming relationships. Hearing loss may contribute to adverse psychosocial outcomes early in life and morbidity in adulthood. Several factors protect against adverse outcomes related to hearing loss, including early intervention, perceived support, and closely knit social networks (Hay-McCutcheon et al., 2018; White et al., 2023). Social support is associated with improved outcomes after auditory rehabilitation and may influence neural plasticity after cochlear implantation (Tang et al., 2017). Finally, hearing loss may serve as a modifiable risk factor for comorbidities, such as Alzheimer’s disease and dementia, with few preventative or therapeutic options. The social neuroscience underlying hearing loss is a rich, yet underexplored, field of study, and developing social interventional tools may have profound implications on a broad range of health outcomes (Saxena et al., 2015; Hohmann, 2023).

## Author contributions

AP and AD conceived of the review. All authors contributed to the outline and literature review. AP, NJ, MK, and AD participated in manuscript writing. All authors performed revisions and approved the final submission.
